# Comparative Study of Microstructural Characteristics and Hardness of β-Quenched Zr702 and Zr–2.5Nb Alloys

**DOI:** 10.3390/ma12223752

**Published:** 2019-11-14

**Authors:** Jiahong Dai, Haotian Guan, Linjiang Chai, Kang Xiang, Yufan Zhu, Risheng Qiu, Ning Guo, Yuanzhuo Liu

**Affiliations:** 1 College of Materials Science and Engineering, Yangtze Normal University, Chongqing 408100, China; 2 College of Materials Science and Engineering, Chongqing University of Technology, Chongqing 400054, China; 3 College of Materials Science and Engineering, Chongqing University, Chongqing 400044, China; 4 Faculty of Materials and Energy, Southwest University, Chongqing 400715, China

**Keywords:** Zr alloy, β quenching, phase transformation, grain refinement, electron backscatter diffraction

## Abstract

In this study, two commercial Zr alloys (Zr702 and Zr–2.5Nb) were subjected to the same β-quenching treatment (water cooling after annealing at 1000 °C for 10 min). Their microstructural characteristics and hardness before and after the heat treatment were well characterized and compared by electron channel contrast (ECC) imaging, electron backscatter diffraction (EBSD) techniques, and microhardness measurements. Results show that after the β quenching, prior equiaxed grains in Zr702 are transformed into Widmanstätten plate structures (the average width ~0.8 μm) with many fine precipitates distributed along their boundaries, while the initial dual-phase (α + β) microstructure in Zr–2.5Nb is fully replaced by fine twinned martensitic plates (the average width ~0.31 μm). Differences in alloying elements (especially Nb) between Zr702 and Zr–2.5Nb are demonstrated to play a key role in determining their phase transformation behaviors during the β quenching. Analyses on crystallographic orientations show that the Burgers orientation relationship is well obeyed in both the alloys with misorientation angles between α plates essentially focused on ~60°. After β quenching, the hardnesses of both alloys were increased by ~35%–40%. Quantitative analyses using the Hall–Petch equation suggest that such an increase was mainly attributable to phase transformation-induced grain refinements. Since Nb is able to effectively refine the β-quenched structures, a higher hardness increment is produced in Zr–2.5Nb than in Zr702.

## 1. Introduction

Zirconium (Zr) and its alloys have wide structural applications in biomedical, chemical, and nuclear industries due to their excellent biocompatibility, good corrosion resistance, and low thermal neutron absorption [1,2,3,4,5]. Pure Zr has a hexagonal close-packed (hcp) structure (α-Zr) at room temperature. At ~865 °C, it will be transformed into β-Zr with a body-centered cubic (bcc) structure [6]. The transformation between the α and β phases is known to be related by the Burgers orientation relationship (OR): {0001}_ɑ_∥{110}_β_ and <11-20>_ɑ_∥<111>_β_ [7]. With respect to commercially used Zr alloys, they can be classified into two major categories of Nb-free and Nb-bearing because of the important role that can be played by Nb in affecting their microstructures and properties. After typical fabrications, the latter (like Zr–2.5Nb) exhibits a dual-phase (α + β) microstructure at room temperature while β-Zr is usually absent in the former (such as Zircaloy-2/4 and Zr702) [8]. In practice, in order to reach good homogenization of alloying elements and/or optimized microstructures, β quenching is often carried out for various Zr alloys before rolling and annealing treatments [9,10,11]. In view of the importance of the β treatments, the effects of temperatures, time, and cooling rates on their β-cooled microstructures have been investigated by different researchers [12,13,14,15,16,17,18,19,20,21,22]. For example, Crepin et al. [12] studied morphology and compositions of the particles precipitated in Zr702 after slow β cooling. Massih et al. [14,16] tried to quantify the correlation between α-plate widths and β cooling rates in Zircaloy-2. Detailed microstructural features of martensites were well characterized by electronic microscopy in Zircaloy-4 [19] and Zr–xNb alloys [13,15]. Crystallographic orientations of various plate structures induced by β cooling in a few Zr alloys were also tentatively explored in [17,18,20].

Nevertheless, it is noted that dedicated comparisons between β-quenched microstructural characteristics of the Nb-free and the Nb-bearing Zr alloys are seldom conducted, leading to incomplete understanding of their microstructural differences. Such work is believed to not only help in good understanding of the property variations induced by β quenching in both types of Zr alloys but may also provide important implications for optimizing their processing parameters and alloy compositions.

Hence, in the present work, two typical commercial Zr alloys (Zr702 and Zr–2.5Nb) were subjected to the same β quenching. Their microstructural characteristics before and after heat treatment were carefully characterized and compared by employing electron channel contrast (ECC) imaging and electron backscatter diffraction (EBSD) techniques. Subsequently, specimen hardnesses and their correlation with microstructural characteristics were examined to explore reasons for their variations. Although rapid β cooling is practically effective in refining grains and bringing hardening in both types of Zr alloy, the results and analyses presented in this study demonstrate that such effects can be greatly intensified by the addition of Nb.

## 2. Experimental 

The as-received materials were a recrystallization annealed Zr702 sheet and a forged Zr–2.5Nb rod, with their chemical compositions shown in Table 1. Rectangular specimens with dimensions of 12, 10, and 2 mm along rolling, transverse and normal directions (RD, TD, and ND) were cut from the as-received Zr702 sheet. From the Zr–2.5Nb material, specimens of 10, 8, and 3 mm in length, width, and height (accordingly denoted as RD, TD, and ND like those of the Zr702 sheet in the following) were machined out. After surface cleaning, these specimens were sealed in quartz tubes under vacuum and heat-treated at 1000 °C (above β transus of both the alloys) for 10 min in a box furnace (SX2-8-16). Subsequently, the quartz tubes were taken out and smashed immediately to allow the specimens to be rapidly quenched into cold water (an estimated cooling rate of ~1000 °C/s).

A field emission gun scanning electron microscope (SEM, Zeiss Sigma HD, Jena, Germany) equipped with a back-scattered electron detector and an EBSD system was utilized to perform microstructural characterization [23,24]. The back-scattered electron detector allowed ECC images to be taken for direct microstructural observation. The EBSD system consisted of an Oxford Instruments NordlysMax^2^ detector, with Aztec 2.4 software for orientation information acquisition and Channel 5 software for data post-processing, respectively. During the EBSD examinations, a scanning area on the RD-ND plane was examined for each specimen at step sizes of 40–200 nm. Local compositions of the specimens were detected by an energy dispersive spectroscope (EDS) attached to the SEM. In addition, a FEI Tecnai G2 F20 transmission electron microscope (TEM, Hillsboro, OR, USA) was employed for revealing specific internal structures in the β-quenched specimens. Hardness measurements were performed for all specimens using a Vickers indenter (Everone MH-5L, Shanghai, China) at a load of 490 mN, with at least twelve measurements made for each specimen to calculate an average value. Prior to SEM microstructural examinations, the to-be-analyzed surfaces were ground by SiC abrasive paper and then electro-polished in a mixed solution of 10 vol.% perchloric acid, 20 vol.% butyl cellosolve and 70 vol.% methanol at 20 V and −30 °C for 30–60 s. Specimens for TEM observations were prepared by a twin-jet electropolisher using a mixed solution of 10 vol.% perchloric acid and 90 vol.% alcohol and at 30 V and −30 °C for ~90 s.

## 3. Results

### 3.1. Microstructural Characteristics 

**Figure 1:**
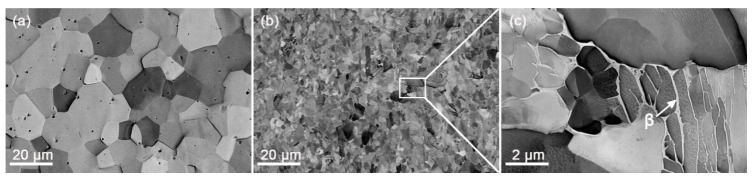
ECC images of the as-received (**a**) Zr702 and (**b**) Zr–2.5Nb materials; (**c**) magnified observation of the boxed region in (**b**) with the white arrow indicating β-Zr.

Figure 1 shows ECC images of the as-received materials (Zr702 and Zr–2.5Nb). From Figure 1a, it can be observed that the starting microstructure of the Zr702 material is composed of equiaxed grains with relatively uniform sizes, suggesting sufficient recrystallization [25,26]. By use of the linear intercept method, its average grain size is measured to be ~8.3 μm. As indicated by the white arrows in Figure 1a, there exist many second phase particles (SPPs) randomly distributed inside grains or along their boundaries. EDS analyses suggest that the SPPs contain Zr, Fe, and Cr, which should correspond to Zr(Fe,Cr)_2_ Laves phases commonly found in many commercial Zr alloys [27]. From Figure 1b, it can be seen that grains in the as-received Zr–2.5Nb material are much smaller than in Zr702, with an average grain size measured to be ~1.4 μm. A boxed region in Figure 1b is magnified in Figure 1c, from which a dual-phase microstructure can be clearly revealed, i.e., equiaxed or plate-shaped α grains surrounded by thin β films (Nb-enriched) [28].

**Figure 2:**
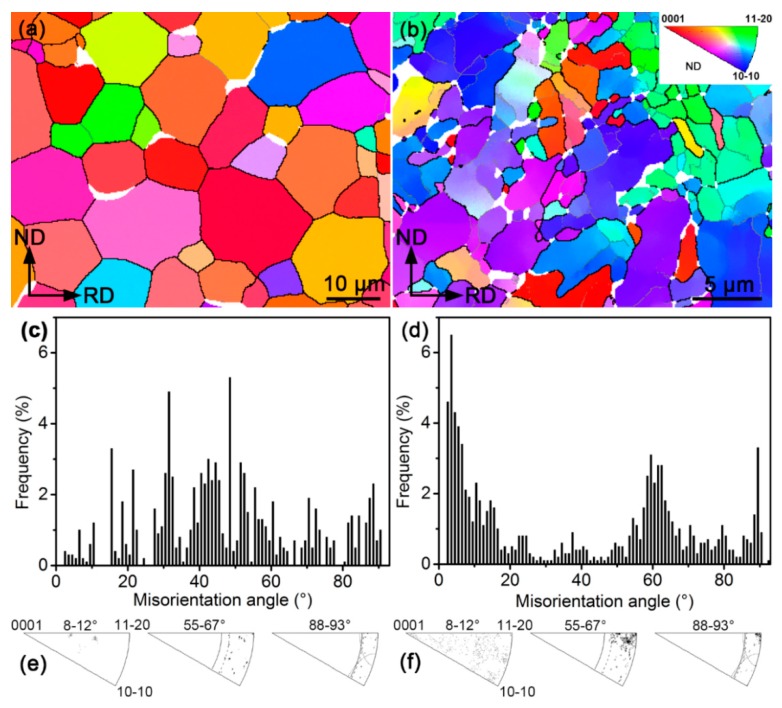
(**a**) EBSD IPF map (black and gray lines indicating HABs and LABs, respectively), (**c**) misorientation angle, and (**e**) rotation axis distributions of the as-received Zr702 specimen; (**b**), (**d**), and (**f**) are accordingly those of the as-received Zr–2.5Nb specimen.

Figure 2 presents features of crystallographic orientations in both the as-received materials revealed by the EBSD. From Figure 2a, a rather uniform orientation (color) can be noted for most grains in the as-received Zr702 and they are essentially separated with each other by high angle boundaries (HABs, θ > 15°). As for the as-received Zr–2.5Nb, Figure 2b reveals that there exist a large number of low angle boundaries (LABs, 2° < θ < 15°) along with the HABs. Meanwhile, orientations inside some grains are found to be heterogeneous, suggesting the existence of orientation gradients. As revealed in Figure 2c,e, misorientation angle and rotation axis distributions of the as-received Zr702 are highly scattered. By contrast, three evident misorientation angle peaks can be observed around 3–5, 60, and 90° for the as-received Zr–2.5Nb (Figure 2d). It is further revealed in Figure 2f that rotation axes corresponding to the two peaks around 60 and 90° are relatively concentrated, consistent with typical Burgers misorientation characteristics [29].

**Figure 3:**
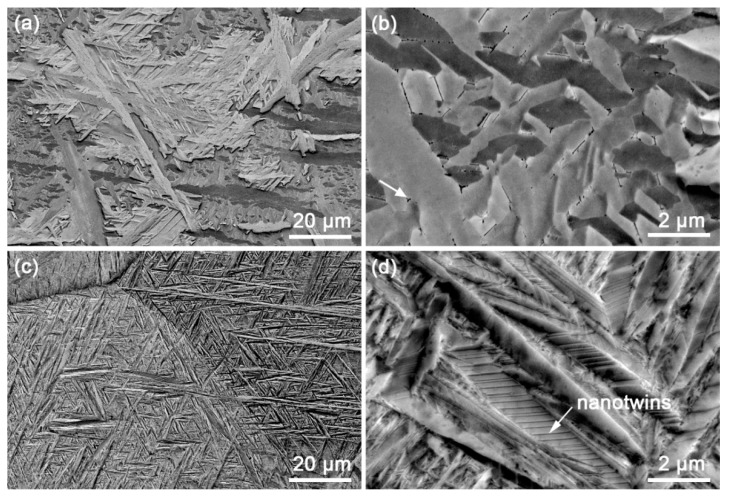
ECC images of the β-quenched specimens: (**a**,**b**) Zr702 and (**c**,**d**) Zr–2.5Nb; arrows in (**b**) and (**d**) indicate precipitates and nanotwins, respectively.

**Figure 4:**
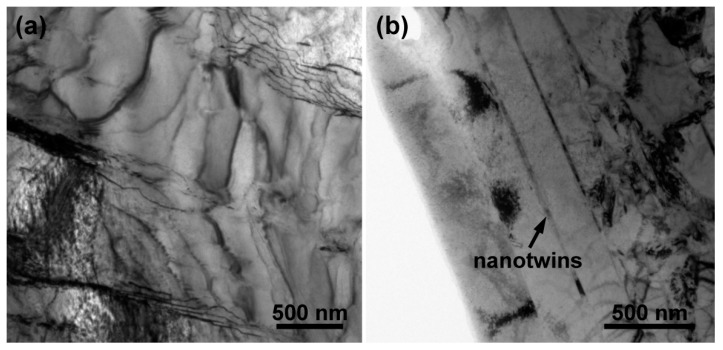
TEM bright-field images of the β-quenched specimens: (**a**) Zr702 and (**b**) Zr–2.5Nb.

Figure 3 presents low- and high-magnification ECC images of the β-quenched specimens. Figure 3a shows that the prior equiaxed grains in Zr702 seem to be fully replaced by parallel or intersected plate structures after the β quenching. It is more clearly revealed in Figure 3b that widths of most plates are <1 μm with their average value measured to be ~0.8 μm. Also, from Figure 3b, dense small precipitates are found to exist along plate boundaries and EDS analyses indicate that they are also comprised of Zr, Fe, and Cr. This suggests that the precipitation of alloying elements in Zr702 is not completely suppressed during the water cooling in spite of a relatively high cooling rate [30]. After comparing with results reported in literature [16,31], the microstructural features presented in the β-quenched Zr702 are confirmed to be Widmanstätten structures as a result of diffusional β→α phase transformation. Figure 3c shows that the β-quenched Zr–2.5Nb is also composed of parallel or intersected plate structures but with smaller sizes than in Zr702 (Figure 3a). A further observation in Figure 3d reveals a high density of fine twinning lamellae inside the plates, which are absent in the β-quenched Zr702 (Figure 3b). The average plate width in the β-quenched Zr–2.5Nb measured to be ~0.3 μm while the average thickness of twinning lamellae is only ~35 nm. In addition, different from the presence of many precipitates in the Widmanstätten structures (Figure 3b), they are not observed in the β-quenched Zr–2.5Nb (Figure 3d). This suggests that diffusion behaviors of alloying elements are completely suppressed during the β water cooling in Zr–2.5Nb and martensitic β→α transformation occurs [32]. Further observations on the plate structures by TEM are presented in Figure 4. For both the β-quenched Zr alloys, a relatively low dislocation density can be noticed, which was believed to be related to specific strain conditions associated with the β→α phase transformation of Zr [15].

**Figure 5:**
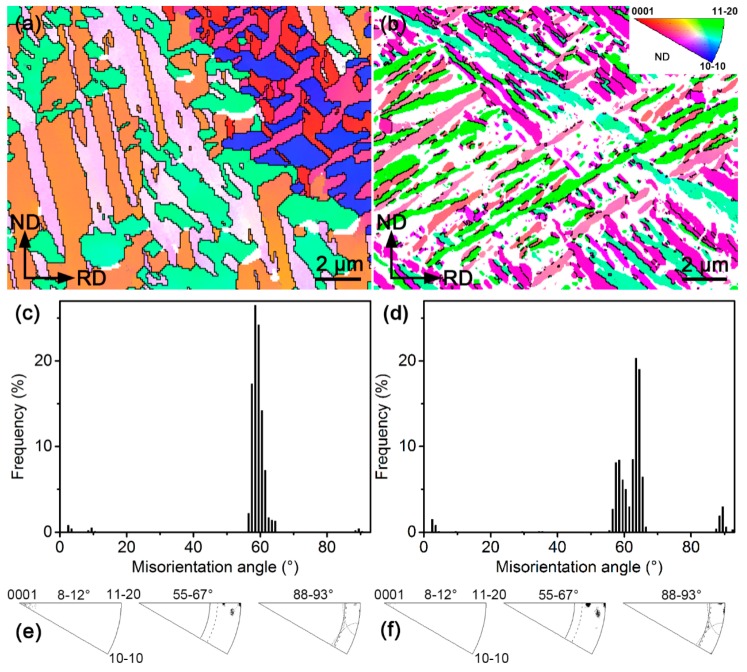
(**a**) EBSD IPF map (black and gray lines indicating HABs and LABs, respectively), (**c**) misorientation angle distribution histogram and (**e**) rotation axis distribution of the β-quenched Zr702 specimen; (**b**), (**d**), and (**f**) are accordingly those of the β-quenched Zr–2.5Nb specimen.

To better probe orientation characteristics of the β-quenched microstructures, their EBSD characterization results are displayed in Figure 5. From Figure 5a,b, one can see that grain (plate) orientations in the β-quenched specimens are more scattered than those in the as-received materials (Figure 2a,b), indicating that many new orientations are produced during the phase transformation. In both the β-quenched specimens, the orientation (color) seems to be rather uniform inside each plate. Most of these plates are separated by HABs and there only exist very few LABs. After comparing Figure 5a,b, one can also confirm that the plate widths of β-quenched structures in Zr–2.5Nb are markedly smaller than in Zr702, consistent with the ECC observations (Figure 3b,d). It is to be noted that the nanotwins in the β-quenched Zr–2.5Nb revealed by the ECC observation (Figure 3d) are not well resolved by the EBSD (Figure 5b) due to their extremely small sizes. Figure 5c,d show similar misorientation angle distribution characteristics (peaked around 60°) for both the β-quenched Zr702 and Zr–2.5Nb, which largely differ from those of the initial specimens (Figure 2e,f). Further analyses in Figure 5e,f show that their misorientation angle peaks exclusively correspond to three rotation axes, which are coincident with those of three Burgers misorientations near 60°. This suggests that the Burgers OR is well obeyed in both the Zr alloys during the β water cooling [7,29].

### 3.2. Hardness Measurements

**Figure 6:**
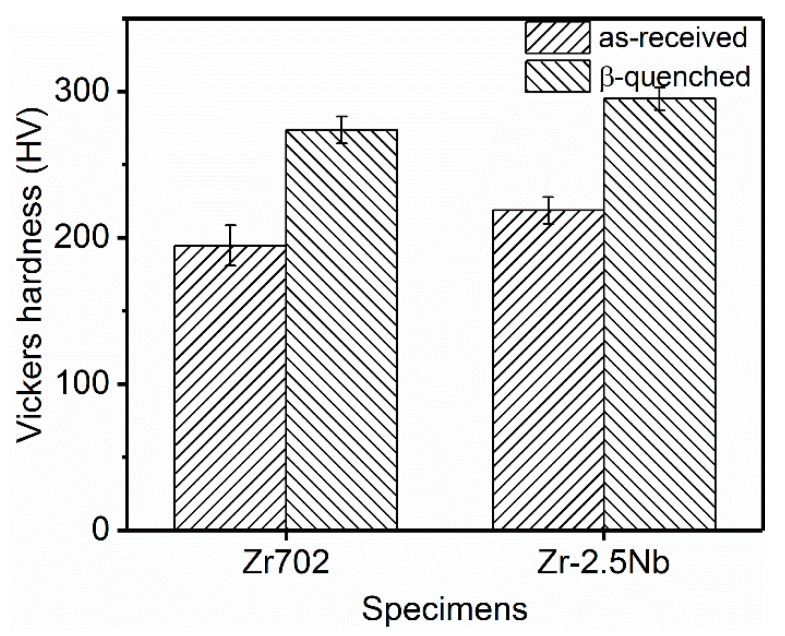
Hardnesses of the as-received and the β-quenched specimens.

Measured hardnesses of various specimens are displayed in Figure 6. After β quenching, the average hardness of Zr702 increases from ~194.8 to ~273.8 HV, (~41% increment) while that of Zr–2.5Nb increases from ~218.8 to ~295.2 HV (~35% increment). The hardness differences between both the Zr alloys and the hardness variations after the heat treatment must be closely related to their specific microstructural characteristics, which will be discussed in detail in the following.

## 4. Discussion

### 4.1. Microstructural Differences between β-Quenched Zr702 and Zr–2.5Nb

Based on the above results, one can confirm that significant differences exist between microstructures of Zr702 and Zr–2.5Nb after the same β-quenching treatment. For the Zr702 specimen, the diffusion of alloying elements occurs during the water cooling, leading the formation of Widmanstätten structures with precipitates distributed along plate boundaries. By contrast, the β-quenched microstructure in Zr–2.5Nb corresponds to twinned martensite resulted from diffusionless transformation. Regarding compositions, Table 1 reveals that the Zr702 material contains 1.15 wt.% Hf but no Nb, while 2.5 wt.% Nb without Hf is added in the Zr–2.5 Nb alloy. Since Hf is in the same group and has the same number of valence electrons as Zr, a small amount of addition could hardly affect phase transformation behaviors of Zr [33]. By contrast, Nb is a typical β-Zr stabilizing element and its addition would effectively decrease the starting temperature of martensitic transformation (*M*_s_) in Zr alloys [34]. Jeong et al. [35] studied β-quenched microstructures in Zr–xNb (x = 0.5–3 wt.%) and found that more added Nb in Zr alloys enabled martensitic transformation to occur at lower cooling rates and led to further refined plate structures. Srivastava et al. [15] figured out that with the Nb content increased from 0.4 to 2.5 wt.% in Zr alloys, β-quenched structures (martensite) changed from dislocated laths to twinned plates with the twins corresponding to {10-11} twinning. One can also note in Table 1 that the Zr702 material contains more O (α-Zr stabilizer [34]) than the Zr–2.5Nb. Woo and Tangri [19] pointed out that more O added into Zr promoted the formation of coarse Widmanstätten plate structures during the β→α transformation and significantly increased the critical cooling rate required for triggering martensitic transformation. It is thus known that Nb has played an important role in Zr–2.5Nb during β water cooling to produce the fine twinned martensite. On the contrary, the absence of Nb along with the higher O content facilitates the formation of Widmanstätten structures in Zr702. It is also noted that both Fe and Cr have rather low solid solubilities (<150 wppm [36]) but relatively high diffusion coefficients in α-Zr [37,38]. This explains why the relatively low additions (~750 wppm) of Fe and Cr in the Zr702 material allow the precipitation of Fe/Cr-bearing particles during the β water cooling, as shown in Figure 3b.

After the β quenching, grain orientations and misorientation angle distributions histograms in both the Zr alloys are distinctly different from those of the as-received specimens. According to the Burgers OR, one β-Zr orientation is able to give birth to twelve different α-Zr orientations (variants) during cooling after considering their crystallographic symmetry. It has been found that variant selection often occurs during the β→α transformation, leading to the appearance of only a few out of all the twelve α variants [9,20,39]. Nevertheless, a recent study by Chai et al. [40] demonstrated that such variant selection behavior could be largely suppressed at high cooling rates (for example water cooling). In the present study, one can expect that the β water cooling should be able to produce many new α orientations in both the Zr alloys, resulting in their grain orientations markedly different from those in the as-received specimens. In addition, the Burgers OR also predicts five possible misorientations between different α variants generated by the same β orientation, namely 10.5°/<0001>, 60°/<11-20>, 60.8°/<34-71>, 63.3°/<5 5 -10 3> and 90°/<34-70> (<34-71> and <34-70> are approximated integer indices of <1 1.38 −2.38 0.36> and <1 1.38 −2.38 0>, respectively, as reported in literature) [41]. Assuming no variant selection, the total frequency of the three misorientations around 60° can reach ~70% [42]. The β-quenched Zr702 and Zr–2.5Nb present very similar misorientation angle distribution characteristics focused on ~60° (Figure 5e,f), with their proportion >90%. This implies that the variant selection may have still occurred to some extent in both of them during the β→α water cooling. 

### 4.2. Correlation between Hardness and Microstructural Characteristics

Hardnesses of both the alloys are increased significantly after the β quenching (Figure 6). In terms of the microstructural characteristics above revealed, specific reasons accounting for such hardness variation can be analyzed. Compared to grain sizes of the as-received materials, the plate widths in both the β-quenched specimens are greatly reduced (Table 2), with most plates separated with each other by HABs. Clearly, the hardness increase can first be attributed to the grain refinement and their contributions can be quantitatively estimated by the Hall–Petch (H-P) equation,

(1) $$\Delta HV_{HP}=k_{HV}(d_{\beta Q}^{-0.5}-d_{AR}^{-0.5}),$$

$\Delta HV_{HP}$ is the hardness increment, $k_{HV}$ is the H-P slope, $d_{AR}$ and $d_{\beta Q}$ are the average grain sizes (plate widths) before and after the β quenching, respectively. At room temperature, the H-P slope of pure α-Zr is ~83.3 HV μm^0.5^ [43]. This value is used to approximate the present cases due to no accurate H-P slopes found for the two dilute Zr alloys employed in this work. The calculated results are listed in Table 2. As for the β-quenched Zr702, the hardness increment calculated using the H-P equation is determined to be ~70.0 HV, close to the hardness difference measured experimentally (~79.0 HV). This suggests that the grain refinement should have made the major contribution to the hardness increase in the β-quenched Zr702. In addition, in spite of the precipitation occurring during the β quenching, the rapid cooling should still be able to solid-solutionize some elements (Fe and Cr) in α plates [30]. As a result, a solid-solution strengthening/hardening effect can be expected [44], which may account for the difference (79 HV − 70 HV = 9 HV) between the hardness increment experimentally measured and that calculated using the H-P equation.

For the β-quenched Zr–2.5Nb, due to more remarkable grain refinement than Zr702, the strengthening/hardening effect associated with grain boundaries is more significant (Table 2). The hardness increment calculated using the H-P equation is ~82.4 HV, slightly higher than the measured value (~76.4 HV). Different from the case in the as-received Zr702, the as-received Zr–2.5Nb possesses a dual-phase (α + β) microstructure (α-Zr grains enclosed by thin β-Zr films (Figure 1c)). The existence of these thin β films was reported to be able to significantly affect dislocation activities inside α grains [45,46] and harden the alloy. Moreover, there are dense LABs in the as-received Zr–2.5Nb (Figure 2d), which would also contribute to hardening [47]. After the β quenching, however, the preexisting β phases disappear completely (Figure 3c,d) and the density of LABs decreases greatly (Figure 5d), necessarily leading to some “softening” effect. This may explain why the hardness increment experimentally measured is slightly lower than that calculated using the H-P equation in Zr–2.5Nb. In addition to the grain refinement, the solid solution of alloying elements (especially Nb) [44] and the presence of nanotwins [48] in the β-quenched Zr–2.5Nb are also hardening contributors. Nevertheless, the above analyses allow us to deduce that such additional hardening will be essentially offset by the softening due to the disappearance of β films and the reduced LABs. Similar to the case in the β-quenched Zr702, the major contribution to hardening in the β-quenched Zr–2.5Nb should also be from the grain refinement.

## 5. Conclusions

(1) After the same β quenching, the prior equiaxed grains in Zr702 are transformed into Widmanstätten plate structures (the average width ~0.8 μm) with many fine precipitates distributed along their boundaries, while the initial α + β dual-phase microstructure in Zr–2.5Nb is fully replaced by fine twinned martensitic plates (the average width ~0.3 μm). Such differences can mainly be attributed to different roles played by specific alloying elements (such as Nb) in determining their phase transformation behaviors. 

(2) In both Zr702 and Zr–2.5Nb, the Burgers OR is well obeyed during the β quenching with misorientation angles between α plates focused on ~60°.

(3) After the β quenching, hardnesses of both the alloys are increased by ~35%–40%, which can mainly be attributed to phase transformation-induced grain refinements. Since the Nb is able to effectively refine the β-quenched structures, a higher hardness increment is produced in Zr–2.5Nb than in Zr702.

## Figures and Tables

**Table 1:** Chemical compositions of the experimental materials (wt.%).

Alloy	Hf	Nb	Fe	Cr	O	Zr
Zr702	1.15	-	0.07	~0.01	0.15	Bal.
Zr–2.5Nb	-	2.50	<0.10	-	0.10	Bal.

**Table 2:** Hardness variations and contributions from the grain refinement.

Alloy	Zr702		Zr–2.5Nb	
	As-Received	β-Quenched	As-Received	β-Quenched
Measured hardness, *H* (HV)	194.8	273.8	218.8	295.2
β-quenching induced hardness increment, Δ*H* (HV)	-	**79.0**	-	**76.4**
Grain size, *d* (μm)	8.3	0.8	1.4	0.3
Hardness contribution calculated using H-P equation, *H_HP_* (HV)	30.1	100.1	73.3	155.7
Hardness increment contributed from β-quenching induced grain refinement, Δ*H_HP_* (HV)	-	**70.0**	-	**82.4**
